# Aerosol, chemical and physical properties of dry powder synthetic lung surfactant for noninvasive treatment of neonatal respiratory distress syndrome

**DOI:** 10.1038/s41598-021-95999-0

**Published:** 2021-08-12

**Authors:** Frans J. Walther, Holly Chan, Jacob R. Smith, Mike Tauber, Alan J. Waring

**Affiliations:** 1 Department of Pediatrics, David Geffen School of Medicine, University of California Los Angeles, Los Angeles, CA 90095 USA; 2 Lundquist Institute for Biomedical Innovation at Harbor-UCLA Medical Center, Torrance, CA 90502 USA; 3 Acorda Therapeutics Inc., Waltham, MA 02451 USA; 4 Department of Medicine, David Geffen School of Medicine, University of California Los Angeles, Los Angeles, CA 90095 USA

**Keywords:** Biochemistry, Biophysics, Biotechnology, Medical research

## Abstract

Inhalation of dry powder synthetic lung surfactant may assist spontaneous breathing by providing noninvasive surfactant therapy for premature infants supported with nasal continuous positive airway pressure. Surfactant was formulated using spray-drying with different phospholipid compositions (70 or 80 total weight% and 7:3 or 4:1 DPPC:POPG ratios), a surfactant protein B peptide analog (KL4, Super Mini-B, or B-YL), and Lactose or Trehalose as excipient. KL4 surfactant underperformed on initial adsorption and surface activity at captive bubble surfactometry. Spray-drying had no effect on the chemical composition of Super Mini-B and B-YL peptides and surfactant with these peptides had excellent surface activity with particle sizes and fine particle fractions that were well within the margins for respiratory particles and similar solid-state properties. Prolonged exposure of the dry powder surfactants with lactose as excipient to 40 °C and 75% humidity negatively affected hysteresis during dynamic cycling in the captive bubble surfactometer. Dry powder synthetic lung surfactants with 70% phospholipids (DPPC and POPG at a 7:3 ratio), 25% trehalose and 3% of SMB or B-YL showed excellent surface activity and good short-term stability, thereby qualifying them for potential clinical use in premature infants.

## Introduction

Surfactant deficiency and lung immaturity are the main causes of respiratory failure in premature infants. Mortality and morbidity of this neonatal respiratory distress syndrome has improved tremendously by the clinical introduction of animal-derived lung surfactant preparations^1^. These surfactants consist of a mixture of phospholipids, especially dipalmitoylphosphatidylcholine (DPPC) and phosphatidylglycerol (PG), and the hydrophobic surfactant proteins B and C (SP-B and SP-C).

**Figure 1:**
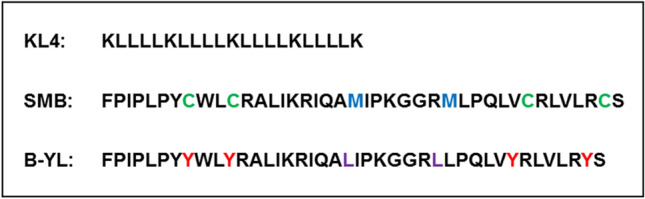
Amino acid composition of surfactant protein B (SP-B) peptide mimics KL4, Super Mini-B (SMB) and B-YL.

Current research has focused on the design and development of a fully synthetic lung surfactant^2^, in which the essential function of native SP-B has been taken over by SP-B and SP-C peptide mimics, e.g., KL4^3^, Mini-B (MB)^4^, Super Mini-B (SMB^5,6^), and B-YL^7^ for native SP-B and SP-C33^8,9^ and SP-Css ion-lock 1^10^ for native SP-C. KL4 is a 21-amino acid peptide with repeating subunits of one lysine (K) and four leucine (L) residues^3^. MB is a 34-amino acid peptide based on the sequence of the N- and C-terminal α-helices of human SP-B and a short loop^4^. In contrast with MB, SMB and B-YL contain an insertion sequence and are 41-amino acid peptides. SMB contains 3 disulfide bridges^5^, whereas B-YL is sulfur-free by replacing the cysteine residues of SMB with tyrosine and less sensitive to oxidation by replacing the methionine residues with leucine^7^ (Fig. 1). The tyrosine for cysteine substitution in B-YL eliminates the need for an oxidation step to secure disulfide linkage in SMB^5–7^, thereby sharply reducing the production costs. Whereas SP-B deficiency through knockout or mutation is lethal in mice and humans^11^, SP-C is less essential than SP-B to maintain lung surfactant activity in vivo^12^.

The primary aim of our study was to develop an advanced dry powder (DP) synthetic lung surfactant for aerosol delivery to preterm infants with respiratory distress syndrome who need noninvasive respiratory support with nasal (continuous positive airway pressure) CPAP in a low technical setting. This surfactant would need to replicate the surface activity of clinically used liquid animal-derived lung surfactant and generate a physically and chemically stable dry powder form that is fit for aerosol delivery. Therefore, we screened various phospholipid quantities (70% and 80% total) and ratios (7:3 and 4:1) of DPPC and palmitoyl-oleyl-PG (POPG), and tested the use of various SP-B peptide mimics (KL4, SMB, B-YL) and stabilizing excipients (Lactose and Trehalose) towards down-selection of a dry powder synthetic lung surfactant for a preclinical trial in surfactant-deficient animal models. Although lactose is an approved excipient for inhaled pharmaceutical products, trehalose was included in the excipient screening procedure as this disaccharide protects labile macromolecules and lipid membranes^13^.

Surface activity of these dry powder surfactant formulations was determined by captive bubble surfactometry (CBS) and integrity of the SP-B peptide mimics by MALDI TOF mass spectrometry. Aerosol and solid-state properties were investigated with particle sizing, X-ray diffraction (XRPD), thermogravimetric analysis (TGA) and differential scanning calorimetry (DSC)^14^. Samples of these surfactant formulations were aged for up to 6 months at 40 °C and 75% humidity and re-tested to determine whether the powders were viable for long-term storage.

## Methods

### Production and formulation of DP synthetic lung surfactant

Dry powder synthetic lung surfactant was designed and formulated at Acorda Therapeutics Inc. utilizing its ARCUS® pulmonary dry powder technology. The peptide mimics of SP-B (KL4, SMB, and B-YL) were synthesized using standard Fmoc protocols, cleaved and purified as detailed previously^3–7,14,15^. Amino acid composition of these peptides is depicted in Fig. 1. Dry powder synthetic lung surfactant was formulated by adding the following components to the organic solvent used for spray drying: DPPC and POPG-Na (Corden Pharma, Liestal, Switzerland); an excipient, i.e., Lactose or Trehalose; NaCl; and a SP-B peptide mimic (KL4, SMB or B-YL). Respirable dry synthetic surfactant particles were produced using a GEA Niro PSD-1 spray dryer (Niro Inc., Copenhagen, Denmark) or Buchi B-290 mini (Buchi Corporation, New Castle, DE 19720) spray dryer. After spray drying, size 00 capsules were filled with surfactant (~ 30 mg per capsule), and packaged in heat-sealable pouches with desiccant. Table 1 describes the composition of the two series of dry powder synthetic lung surfactant formulations studied here.

**Table 1:** Composition of the two series of dry powder synthetic lung surfactants tested.

Surfactant	Formulation	Weight%
**First series**		
PL70[7:3]-SMB	DPPC:POPG:SMB:Lactose:NaCl	49:21:3:25:2
PL70[7:3]-KL4	DPPC:POPG:KL4:Lactose:NaCl	49:21:3:25:2
PL80[7:3]-SMB	DPPC:POPG:SMB:Lactose:NaCl	56:24:3:15:2
PL80[7:3]-KL4	DPPC:POPG:KL4:Lactose:NaCl	56:24:3:15:2
PL80[4:1]-SMB	DPPC:POPG:SMB:Lactose:NaCl	64:16:3:15:2
PL80[4:1]-KL4	DPPC:POPG:KL4:Lactose:NaCl	64:16:3:15:2
**Second series**		
SMB: Lactose	DPPC:POPG:SMB:Lactose:NaCl	49:21:3:25:2
B-YL: Lactose	DPPC:POPG:B-YL:Lactose:NaCl	49:21:3:25:2
SMB: Trehalose	DPPC:POPG:SMB:Trehalose:NaCl	49:21:3:25:2
B-YL: Trehalose	DPPC:POPG:B-YL:Trehalose:NaCl	49:21:3:25:2

### Captive bubble surfactometry

Adsorption and surface tension lowering ability of dry powder synthetic lung surfactant preparations were measured with a captive bubble surfactometer, described and built by Schürch and coworkers^5,16–18^. In this leak-proof system, filled with Goerke’s buffer with 10% sucrose, surfactant floats against a hydrophilic roof consisting of 1% agarose gel. After inserting an air bubble into the chamber, adsorption of the surfactant to the bubble’s air–liquid interface is measured. The bubble chamber is then sealed and quasi-static compression and expansion of the air bubble is done in discrete steps at a rate of 5% of the bubble volume every 10 s for 4–10 cycles. Quasi-static cycling is followed by dynamic compression and expansion cycling between 10 and 110% of the original bubble area at a physiologic cycling rate of 20 cycles/min. Both cycling modalities show extreme flattening of the air bubble in surface-active surfactant preparations. Throughout each experiment, bubble shapes are monitored with continuous video recording and surface tension was analyzed with custom-designed software^19^.

For each measurement, a capsule with ~ 30 mg of dry powder surfactant was opened and its contents were dissolved in 1 ml of distilled water. Two µL of this surfactant sample was then inserted into the bubble chamber, resulting in a surfactant concentration of ~ 50 µg/ml. All measurements were performed in triplicate at 37 °C.

### Chemical stability of surfactant peptide in spray-dried preparations

Matrix-assisted laser desorption/ionization (MALDI) mass spectrometry was applied to determine the integrity of SP-B peptide mimics after exposure to the high temperatures associated with spray drying. One month old spray dried surfactant samples, dispersed in water at the same concentration used for surfactometry, were analyzed using an AB SCIEX TOF/TOF 5800 system (Sciex, Framingham, MA 01701). Spray-dried surfactant peptide-lipid samples containing approximately 50 pmoL of peptide were co-solvated with either ∝ -cyano-4-hydroxycinnamic acid or sinapic acid (10 mg matrix/ml water: acetonitrile 1:1, v:v with 0.3% trifluoroacetic acid) by mixing 24 µl of matrix solution with 1 µl of peptide-lipid dispersion solution co-solvated with an equal volume of acetonitrile with 1% trifluoroacetic acid. 2 µl of this mixture was then deposited onto a metal MALDI sample plate and allowed to air dry before mass spectral measurement. The spectrometer was set for mid-mass positive linear mode detection and the resulting spectrum expressed as the mass/charge ratio (m/z) in daltons (Da). The mass spectra were analyzed using ABI SCIEX Analyst and Data Explorer software.

### Physical stability

Average geometric particle size (gPSD) in microns (d50) was measured using laser diffraction (HELOS, Sympatec, Clausthal-Zellerfeld, Germany). The fine particle fraction (FPF) was measured with a three-stage Andersen Cascade Impactor (ACI-3) operated at a volumetric flow rate of 28.3 and/or 60 l/min (Copley Scientific, Nottingham, UK). The FPF of the total dose was than calculated using 5.8 and 3.3 microns as upper limits at a flow rate of 28.3 l/min. XRPD was used for structural powder characterization with special attention to the characteristic diffraction peak at 21° 2θ that indicates the presence of phospholipids in a bilayer structure. TGA was used to measure the mass loss of a powder sample as a function of temperature and to determine the percentage of volatiles loss up to 120 °C, i.e., residual solvent (ethanol and water). DSC was used to measure temperatures at phase transitions. Low T1 is the characteristic temperature of the first thermal event(s) observed and Low T2 the second set of thermal event(s) observed during a DSC scan at 20 °C/min.

### Statistical analysis

All data are expressed as mean ± standard error of the mean (SEM). Student’s t-tests were used for comparisons of discrete data points and functional data were analyzed with one-way analysis of variance with Tukey’s post-hoc test using SPSS software. Differences with a p value < 0.05 were considered to be statistically significant.

## Results

### Captive bubble surfactometry

**Figure 2:**
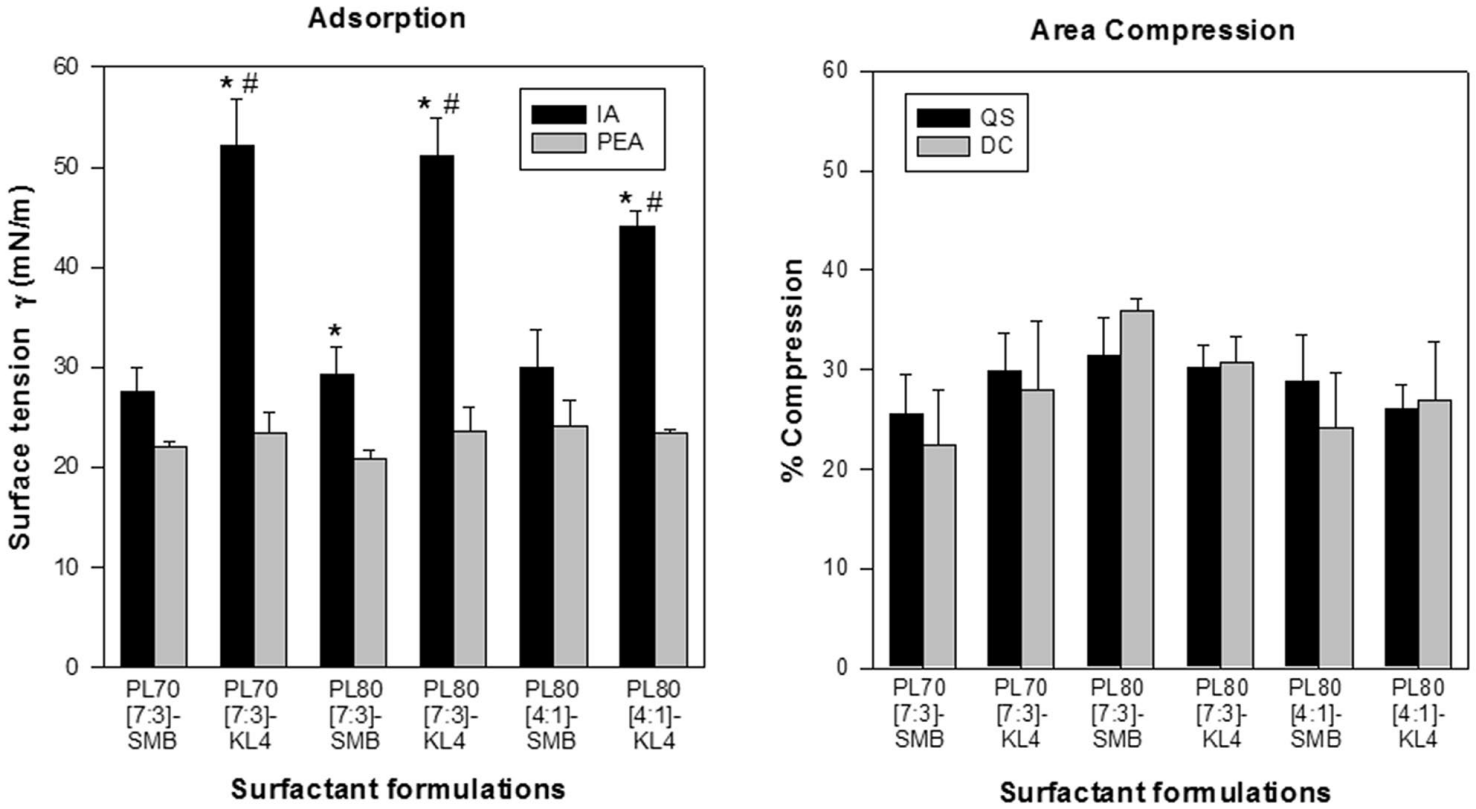
Mean ± SEM adsorption and area compression values of the six dry powder surfactant formulations from the 1st series. Formulations PL70[7:3]-SMB, PL80[7:3]-SMB and PL80[4:1]-SMB contained Super Mini-B (SMB) and formulations PL70[7:3]-KL4, PL80[7:3]-KL4 and PL80[4:1]-Kl4 contained KL4 as surfactant protein B (SP-B) peptide mimic. PL70 and PL80 stand for 70% and 80% phospholipids, [7:3] and [4:1] for DPPC:POPG ratios. The left-sided figure depicts initial adsorption (IA) and post-expansion adsorption (PEA) expressed as surface tension in mN/m. The right-sided figure shows % compression to reach minimum surface tension during quasi-static (QS) and dynamic cycling (DC), i.e. quasi-static cycle 4 and dynamic cycle 10. *p < 0.001 IA vs corresponding PEA values.; ^#^p < 0.02 vs correponding SMB-surfactant. See Table 1 for details on composition of the surfactants.

**Figure 3:**
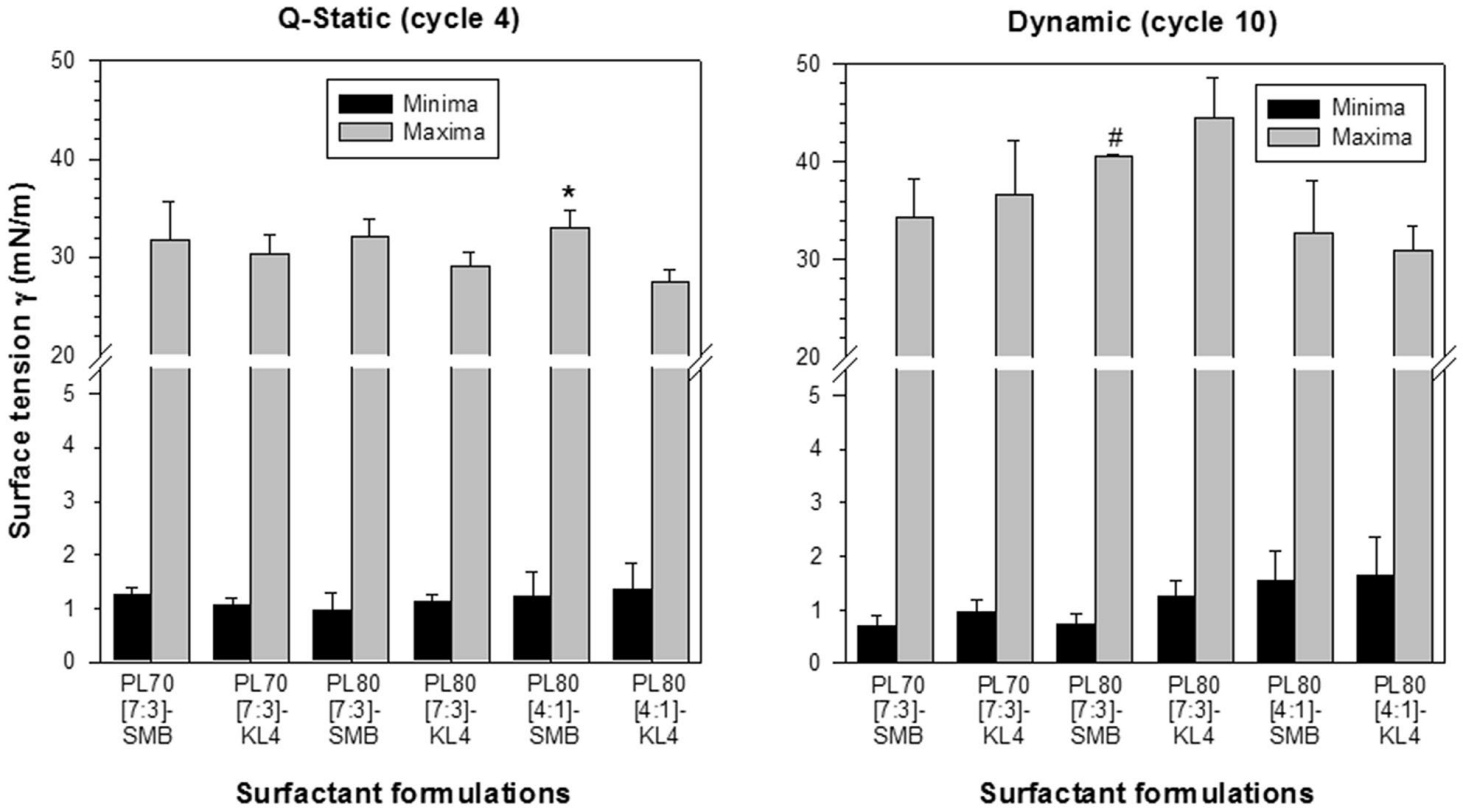
Mean ± SEM quasi-static (cycle 4) and dynamic (cycle 10) cycling data of the six dry powder surfactant preparations from the 1st series at captive bubble surfactometry. Formulations PL70[7:3]-SMB, PL80[7:3]-SMB and PL80[4:1]-SMB contained Super Mini-B (SMB) and formulations PL70[7:3]-KL4, PL80[7:3]-KL4 and PL80[4:1]-Kl4 contained KL4 as surfactant protein B (SP-B) peptide mimic. PL70 and PL80 stand for 70% and 80% phospholipids, [7:3] and [4:1] for DPPC:POPG ratios. Black bars depict minimum surface tension and gray bars maximum surface tension at quasi-static cycle 4 (left-sided figure) and dynamic cycle 10 (right-sided figure) (n = 3–4). *p = 0.04 vs PL80[4:1]-KL4; ^#^p = 0.02 vs PL80[4:1]-KL4. See Table 1 for details on composition of the surfactants.

The first series of six dry powder synthetic lung surfactants focused on phospholipid ratio (DPPC:POPG ratio of 7:3 or 4:1) and quantity (70 or 80 wt%) and SP-B peptide mimic selection (3 weight% of KL4 or SMB), all powders contained lactose as sugar excipient. Initial adsorption (IA) of SMB surfactants was significantly better than that of the KL4 surfactants (p < 0.02) (Fig. 2), but there were no differences in post-expansion adsorption (PEA). IA was higher than PEA for all surfactant formulations, except for PL70[7:3]-SMB and PL80[4:1]-SMB. Average ± SEM minimum surface tension of the three KL4 surfactants during the 1st quasi-static cycle was 5.1 ± 1.7 mN/m versus 1.3 ± 0.3 mN/m in the three SMB surfactants (p = 0.0442), but all surfactants reached average minimum surface tension values < 2 mN/m during the next cycles. Figure 3 shows minimum and maximum surface tension values of these surfactants during quasi-static cycle 4 and dynamic cycle 10. Phospholipid composition (both quantity and ratio) and SP-B peptide mimic selection did not affect minimum surface activity. Average area reduction during compression to reach minimum surface tension varied between 25 and 32% during quasi-static cycle 4 and between 22 and 36% during dynamic cycle 10 (Fig. 2).

**Figure 4:**
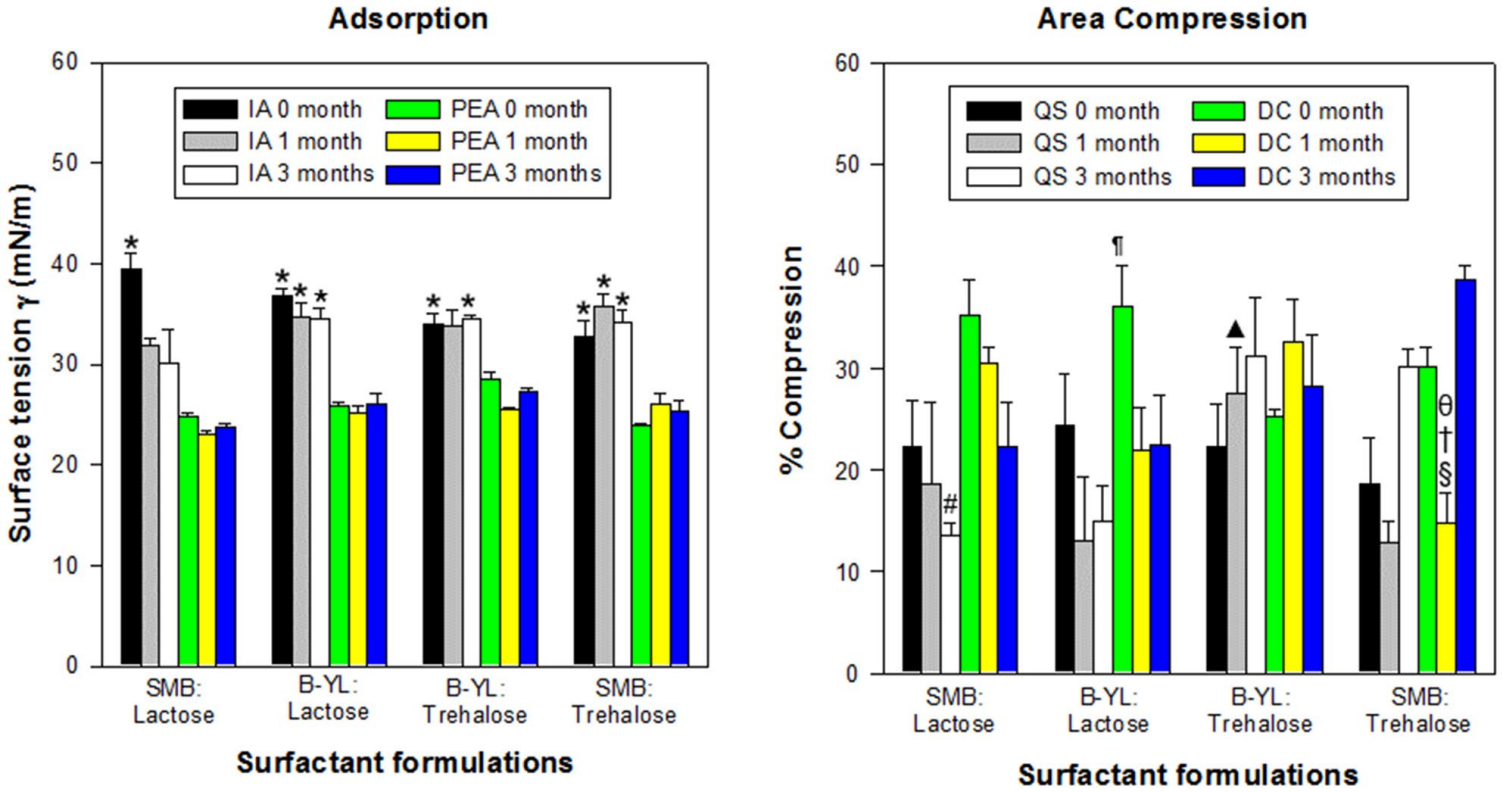
Mean ± SEM adsorption and area compression values of the four dry powder surfactant formulations from the 2nd series. Formulations contained 70% phospholipids with a DPPC:POPG ratio of 7:3, Super Mini-B (SMB) or B-YL as surfactant peptide and lactose or trehalose as excipient. Surfactant formulations were tested at baseline (0 month) and after 1 and 3 months of exposure to 40^0^C and 75% relative humidity (1 month and 3 months). The left-sided figure depicts initial adsorption (IA) and post-expansion adsorption (PEA) expressed as surface tension in mN/m. The right-sided figure shows % compression necessary to reach minimum surface tension during quasi-static (QS) and dynamic cycling (DC) at 0, 1 and 3 months of exposure to 40 °C and 75% relative humidity. *p < 0.001 for IA vs corresponding PEA values.; ^#^p < 0.05 for QS 3 months of SMB: Lactose vs B-YL: Trehalose and SMB: Trehalose; ^▲^p = 0.012 for QS 1 month of B-YL: Trehalose vs SMB: Trehalose; ^§^p < 0.0001 for QS 1 month of SMB: Trehalose vs QS 3 months of SMB: Trehalose; ^¶^p = 0.0254 for DC 0 month of B-YL: Lactose vs B-YL: Trehalose; ^†^p < 0.004 for DC 1 month of SMB: Trehalose vs B-YL:T rehalose and SMB: Lactose; ^θ^p < 0.004 for DC 1 month vs DC 0 and 3 months of SMB-Trehalose. See Table 1 for details on composition of the surfactants.

**Figure 5:**
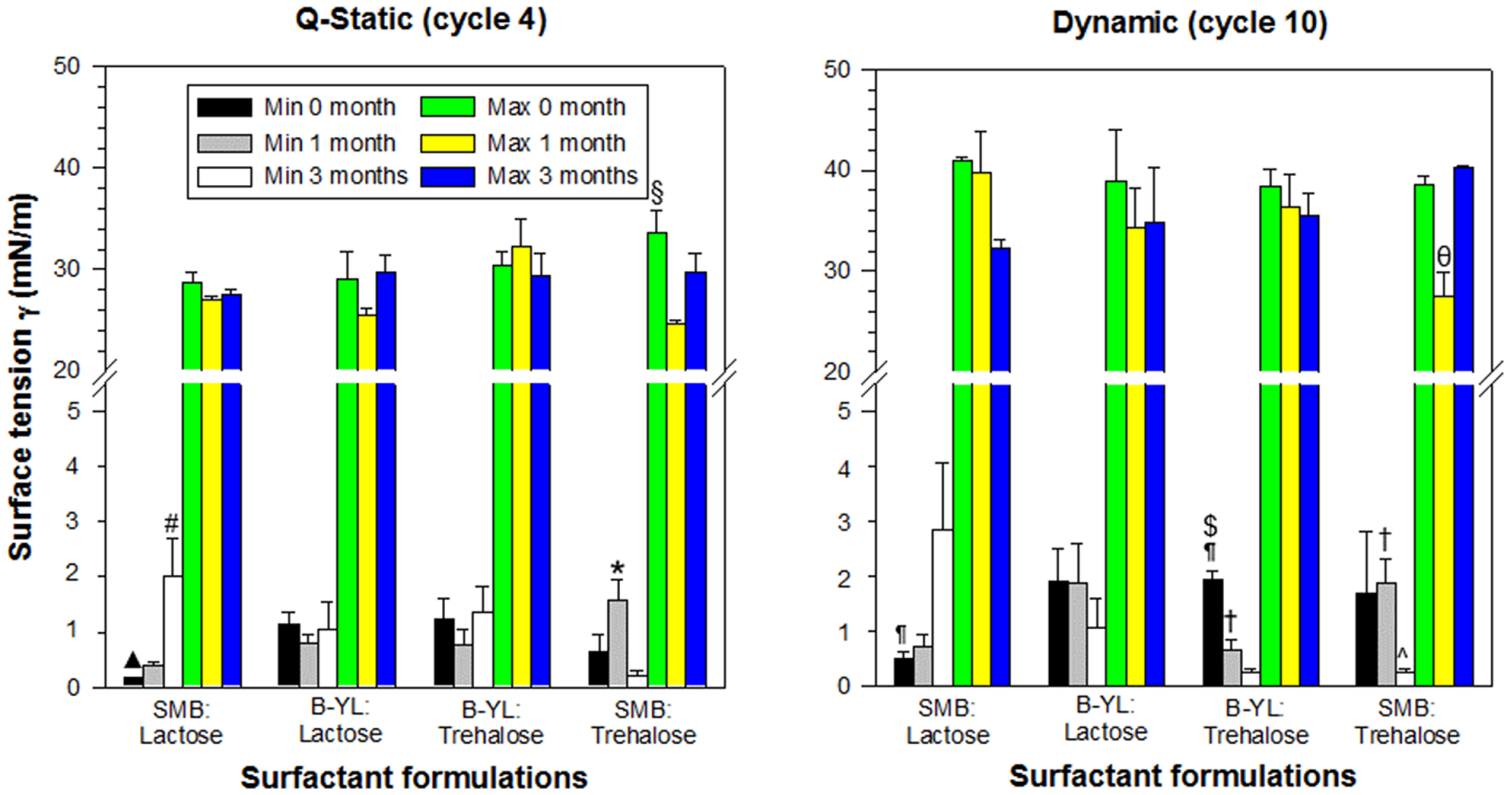
Mean ± SEM quasi-static (cycle 4) and dynamic (cycle 10) cycling data of the four dry powder surfactant preparations from the 2nd series at captive bubble surfactometry. Surfactant formulations included SMB: Lactose, B-YL: Lactose, B-YL: Trehalose and SMB: Trehalose. The left-sided figure depicts minimum and maximum surface tension values at quasi-static cycle 4 and the right-sided figure shows minimum and maximum surface tension values at dynamic cycle 10 after 0, 1 and 3 months of exposure to 40 °C and 75% relative humidity (n = 3–4). Min = mimimum, max = maximum; ^#^p = 0.021 for QS min 0 vs 3 months of SMB: Lactose; ^*^p = 0.024 for QS min 1 vs 3 months for SMB: Trehalose; ^▲^p < 0.02 for QS min 0 month of SMB: Lactose vs B-YL: Lactose and B-YL: Trehalose; ^§^p = 0.017 for QS max 0 vs 3 months of SMB: Trehalose; ^¶^p = 0.003 of DC min 0 months for SMB: Lactose vs B-YL: Trehalose; ^†^p = 0.048 of DC min 1 month of B-YL: Trehalose vs SMB: Trehalose; ^$^p < 0.01 of DC min 0 month vs 1 and 3 months for B-YL: Trehalose; ^^^p < 0.03 of DC min 3 month vs 0 and 1 month for SMB-Trehalose; θ: p < 0.02 of DC max 1 month vs 0 and 3 months for SMB: Trehalose. See Table 1 for details on composition of the surfactants.

Surfactometry of the second series of dry powder synthetic lung surfactant demonstrated that the four dry powder surfactant formulations with 70% phospholipids at a 7:3 DPPC:POPG ratio and 3% of SMB or B-YL as SP-B peptide mimic and 25% of Lactose or Trehalose as excipient were highly surface-active. Surface activity was measured with captive bubble surfactometry shortly after production (0 month) and after 1 and 3 months of exposure to 40 °C and 75% relative humidity (1 month and 3 months). Initial adsorption at 0 month was in general higher than PEA (p < 0.001, Fig. 4). Average minimum surface tension was < 2 mN/m during quasi-static cycle 4 with a maximum surface tension of 29–35 mN/m (Fig. 5). During dynamic cycle 10, all four formulations also reached average minimum surface tension values < 2 mNm and average maximum surface tension varied between 38 and 41 mN/m (Fig. 5). Average area reduction during compression to reach minimum surface tension varied between 19 and 24% during quasi-static cycle 4 and between 26 and 34% during dynamic cycle 10 (Fig. 4). Although % compression was on average higher during dynamic than quasi-static cycling, the differences were not statistically significant.

Exposure to 40 °C and 75% relative humidity for 1 or 3 months did not significantly affect the surface tension lowering capabilities of these four surfactant formulations. After 1 and 3 months of stress exposure, average minimum surface tension values during quasi-static and dynamic cycling were still < 3 mN/m (Fig. 5). However, minimum surface tension at quasi-static cycle 4 of SMB:Trehalose was higher at 1 than at 3 months (p = 0.024) and minimum surface tension of SMB:Lactose was higher at 3 months than at 0 months (p = 0.021) (Fig. 5). Minimum surface tension during dynamic cycling decreased during heat and humidity stress in both Trehalose surfactant formulations (p < 0.03). Maximum surface tension values during dynamic cycling varied between 27 and 45 mN/m (Fig. 5). Area reduction secondary to compression during quasi-static and dynamic cycling varied considerably under stress exposure, except for B-YL:Trehalose surfactant (Fig. 4).

**Figure 6:**
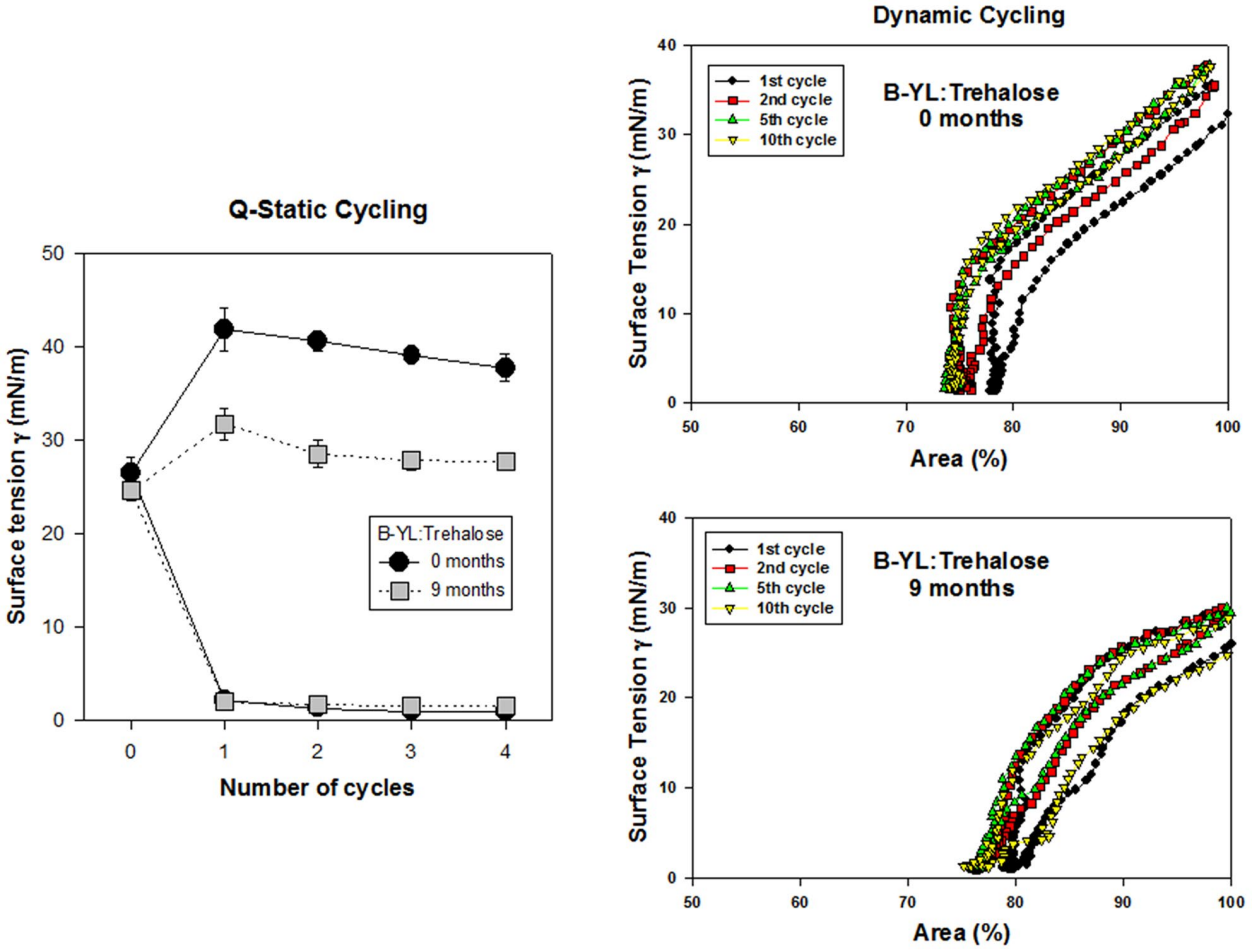
Quasi-static (left) and dynamic cycling (right) isotherms at captive bubble surfactometry of a B-YL: Trehalose surfactant (DPPC:POPG:B-YL:Trehalose:NaCl 49:21:3:25:2) at baseline and after 9 months of exposure to 40 °C and 75% relative humidity. Differences in minimum surface tension at quasi-static cycling and % compression area to reach minimum surface tension at dynamic cycling were not statistically significant.

B-YL:Trehalose surfactant stood out at 1 and 3 months of stress exposure with minimum surface tension values << 2 mN/m during quasi-static and dynamic cycling (Fig. 5). Figure 6 shows that surface activity of a repeat production of B-YL:Trehalose surfactant did not change after aging for 9 months. These values are comparable to those of the clinical porcine surfactant Curosurf®.

### Physical stability

Table 2 summarizes the aerosol and solid-state properties of the first series of dry powder surfactant formulations at baseline and after exposure for 2 weeks or 1 months to 40 °C and 75% relative humidity. gPSD and FPF were well within the margins for respiratory particles at baseline, but the KL4 and SMB powders with 70% phospholipids and 25% lactose (PL70[7:3]-KL4 and PL70[7:3]-SMB) had a more stable FPF than the powders with 80% lipids and only 15% lactose. Solid state properties were similar among the various surfactant formulations. They were semi-crystalline with a characteristic diffraction peak at 21° 2θ, which can be attributed to the presence of phospholipids in a bilayer structure. Residual solvent content (TGA-120) was less than 2.2%. The dry powder formulations contained phase transitions at ~ 38–44 °C, calculated as the intercept of a step transition, and 49–63 °C, calculated as a peak extremum of an endotherm.

**Table 2:** Aerosol properties and solid state properties of each of the first series of dry powder surfactant formulations tested.

Surfactant	Description	Condition		gPSD (µm)	ACI 60 LPM		ACI 28.3 LPM		XRPD	TGA-120%	DSC	
					Size 00 FPF < 5.6 µm (%)	Size 00 FPF < 3.4 µm (%)	Size 00 FPF < 5.6 µm (%)	Size 00 FPF < 3.4 µm (%)			Low T1 (°C)	Low T2 (°C)
PL70[7:3]-SMB	DPPC:POPG:SMB: Lactose:NaCl 49:21:3:25:2	t = 0		5.1	68	48	50	28	SC-D	2.12	40.4	49.3
		40 °C/75% RH	2 week		60	37	34	16	SC-D	1.91	n.c.	61.9
			1 month		55	34	29	12	SC-D	1.94	43.8	51.9
PL70[7:3]-KL4	DPPC:POPG:KL4: Lactose:NaCl 49:21:3:25:2	t = 0		4.5	71	52	60	37	SC-D	2.11	39	50.3
		40 °C/75% RH	2 week		50	34	21	9	SC-D	1.96	43.3	52
			1 month		47	29	18	8	SC-D	2.02	43.9	52.5
PL80[7:3]-SMB	DPPC:POPG:SMB: Lactose:NaCl 56:24:3:15:2	t = 0		5.3	61	41	44	23	SC-D	2.01	38.8	59.1
		40 °C/75% RH	2 week		33	23	10	5	SC-D	1.78	40.9	59.9
			1 month		29	20	9	5	SC-D	1.81	41.7	59.6
PL80[7:3]-KL4	DPPC:POPG:KL4: Lactose:NaCl 56:24:3:15:2	t = 0		5.0	67	48	53	31	SC-D	1.86	39.6	59.5
		40 °C/75% RH	2 week		33	23	7	2	SC-D	1.71	43.2	58.8
			1 month		23	14	7	3	SC-D	1.66	41.8	59.2
PL80[4:1]-SMB	DPPC:POPG:SMB: Lactose:NaCl 64:16:3:15:2	t = 0		5.3	64	42	44	25	SC-D	1.95	40.2	62.5
		40 °C/75% RH	2 week		49	30	1	0	SC-D	1.83	NT**	NT
			1 month		43	28	17	7	SC-D	1.86	41.7	62.9
PL80[4:1]-KL4	DPPC:POPG:KL4: Lactose:NaCl 64:16:3:15:2	t = 0		5.5	66	47	54	29	SC-D	1.87	39.9	62
		40 °C/75% RH	2 week		35	20	1	0	SC-D	1.81	42.4	62
			1 month		27	16	2	0	SC-D	1.82	42.2	62.5

Powders vary according to phospholipid ratio (DPPC:POPG 7:3 or 4:1) and quantity (70 or 80 wt%) and SP-B peptide mimic (SMB or KL4), but all contain lactose as excipient.

*RH: Relative Humidity; **NT: not tested; n.c.: not calculated.

Table 3 provides the same data for the second series of dry powder surfactant formulations at baseline and after exposure for 2 weeks, 1, 3, and 6 months to 40 °C and 75% relative humidity. gPSD and FPF were again well within the margins for respiratory particles. Solid state properties were similar among the various dry powder surfactant formulations that were semi-crystalline with a characteristic diffraction peak at 21° 2θ due to the presence of phospholipids. Residual solvent content was less than 1.5%. The dry powder formulations contained phase transitions at ~ 45–53 °C, calculated as the intercept of a step transition, and 61–67 °C, calculated as a peak extremum of an endotherm.

**Table 3:** Aerosol properties and solid state properties of each of the second series of dry powder surfactant formulations tested.

Surfactant	Description	Condition		gPSD (µm)	Relative to emitted powder		Relative to total powder		XRPD	TGA-120%	DSC	
					ACI 28.3 LPM						Low T1 (^o^C)	Low T2 (^o^C)
					FPF < 5.6 µm (%)	FPF < 3.4 µm (%)	FPF < 5.6 µm (%)	FPF < 3.4 µm (%)				
SMB: Lactose	DPPC:POPG: SMB:Lactose: NaCl 49:21:3:25:2	t = 0		4.2	49.7	30.1	49.3	29.9	SC-D	2.11	40.2	51.5
		40 °C/75% RH**	2 week		53	30	52.5	29.7	SC-D	0.61	n.c.	60.5
			1 month		47.7	28.2	41.7	24.7	NT*			
			3 month		44.8	26.8	44.3	26.5	SC-D	0.98	n.c.	61.7
B-YL: Lactose	DPPC:POPG: B-YL:Lactose: NaCl 49:21:3:25:2	t = 0		3.2	51.1	30.4	48.7	29	SC-D	2.08	38.3	51.3
		40 °C/75% RH	2 week		49.2	26.8	48.8	26.6	SC-D	0.75	n.c.	61.4
			1 month		61.1	38.85	58.1	36.9	NT			
			3 month		54	31.2	50.1	29	SC-D	0.80	n.c.	64.2
			6 month		54.7	30.3	54.2	30	SC-D	0.75	n.c.	63.1
SMB: Trehalose	DPPC:POPG: SMB:Trehalose:NaCl 49:21:3:25:2	t = 0		3.9	39	19.7	38.6	19.5	SC-D	1.58	47.8	58
		40 °C/75% RH	2 week		41.9	19.7	41.3	19.4	NT			
			1 month		40.8	17.9	40.4	17.7				
			3 month		43.6	20.4	43.5	20.3	SC-D	0.83	n.c.	60.4
B-YL: Trehalose	DPPC:POPG: B-YL:Trehalose: NaCl 49:21:3:25:2	t = 0		3.8	42.8	25.1	38.6	19.5	SC-D	1.54	42.1	51.9
		40 °C/75% RH	2 week		42.6	22.2	41.3	19.4	NT			
			1 month		47.9	23.5	40.4	17.7				
			3 month		46	22.4	43.5	20.3	SC-D	0.93	n.c.	59.6
			6 month		41.6	20.4	41	20.2	SC-D	0.91	n.c.	59.3

Powders have the same phospholipid composition, but differ in regard with SP-B peptide mimic (SMB or B-YL) and excipient (Lactose or Trehalose). Samples have desiccant incorporated in the packaging.

*NT: Not Tested; **RH: Relative Humidity; n.c.: not calculated.

### Chemical stability of surfactant peptides in spray-dried preparations

**Figure 7:**
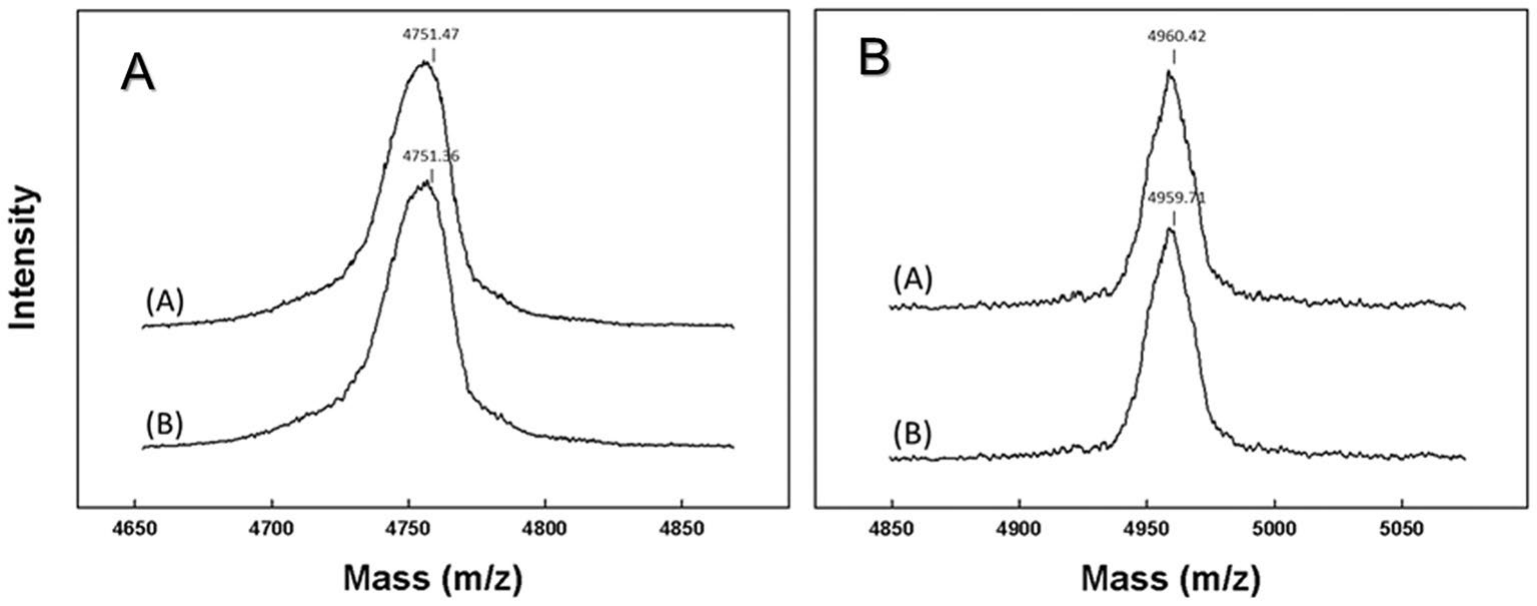
Maldi TOF mass spectra of the four dry powder surfactant preparations from the 2nd series (SMB or B-YL peptide with Lactose or Trehalose as excipient). Figure A on the left: (**A**) SMB: Lactose surfactant (DPPC:POPG:SMB:Lactose:NaCl 49:21:3:25:2) and (**B**) SMB: Trehalose surfactant (DPPC:POPG:SMB:Trehalose:NaCl 49:21:3:25:2). Figure B on the right: (**A**) B-YL: Lactose surfactant (DPPC:POPG:B-YL:Lactose:NaCl 49:21:3:25:2) and (**B**) B-YL: Trehalose (DPPC:POPG:B-YL:Trehalose:NaCl 49:21:3:25:2). Samples had been stored at 5 °C for 1 month before analysis by mass spectrometry. Spray drying during surfactant production did not affect the integrity of the SMB peptide in phospholipid mixtures containing either Trehalose or Lactose as excipient.

MALDI mass spectrometry confirmed the appropriate masses for SMB and B-YL in the second series of dry powder surfactant formulations (Fig. 7). No degradation products of these SP-B peptide mimics were detected in samples stored at 5 °C for 1 month before analysis by mass spectrometry, i.e., spray drying during surfactant production did not affect the integrity of these peptides in phospholipid mixtures containing either Trehalose or Lactose as excipient.

## Discussion

In this study, we investigated the aerosol, chemical and physical properties of two series of dry powder lung surfactant formulations that differed with respect to phospholipid composition, choice of SP-B peptide analog used as active pharmaceutical ingredient (API), and the sugar-based excipient.

The effect of phospholipid composition was investigated using captive bubble surfactometry in the first series of dry powder surfactants. Total quantity (70 or 80 wt%) and ratio (7:3 or 4:1) of DPPC and POPG did not directly affect surface activity, but initial adsorption was significantly higher in surfactant formulations with KL4 as SP-B peptide mimic. Considering the relatively high costs of POPG over DPPC and advancing viscosity at higher concentrations of DPPC, dry powder surfactants in the second series were produced with 70% of phospholipids at a 7:3 DPPC:POPG ratio. This choice was enforced by the finding that a higher phospholipid concentration led to a lower concentration of sugar excipient (15% instead of 25%) and a less stable FPF (Table 2).

In the first series of surfactants, KL4 surfactant underperformed with regard to initial adsorption and surface activity during the first quasi-static cycle when compared to SMB. In the second series of surfactants, surface activity, particle size and FPF, chemical and physical properties of SMB and its sulfur-free derivative B-YL were similar (Table 3). Lower production costs of B-YL peptide by avoiding the oxidation step in the production of SMB therefore warranted a choice for B-YL as lead API.

The choice between lactose and trehalose for sugar excipient was based on the reduction in hysteresis area seen in lactose formulations during dynamic cycling at captive bubble surfactometry after 1 and 3 months of exposure to 40 °C and 75% relative humidity (Fig. 5). These downsides were not found in the SMB:Trehalose and B-YL:Trehalose formulations.

Viability of dry powder surfactant for long-term storage will require further studies. We know that the material may still have some surface activity; however, we also know that there will be chemical degradation over time, especially of the phospholipids^6^. Therefore, it is important to focus on both surface activity and chemical assay/potency after long-term storage.

Limitations of this study included the need to reconstitute the surfactant formulations and the occurrence of overcompression in some dynamic cycling runs. We considered aerosolization of the dry powder surfactants into the sample chamber, but this was not practical as it was not possible to blow the dry powder through a 1 µm needle into the sample chamber filled with 10% Goerke’s sucrose solution. Overcompression is an artefact that can occur because the captive bubble software is set to perform dynamic compression and expansion cycling between 10 and 110% of the original bubble area.

Dry powder synthetic lung surfactant has a 40-year history. Morley et al.^20^ prepared a protein-free dry artificial lung surfactant (ALEC) consisting of DPPC and PG at a ratio of 7:3 and blew it down an endotracheal tube into the lungs of 22 very premature babies at birth. These infants required less ventilatory support in the first six hours and had a lower neonatal mortality than a group of 33 untreated controls. The introduction of intratracheal administration of bovine and porcine lung surfactant suspensions in the 1980s quickly overshadowed the early work with protein-free dry powder surfactant formulations^21^. Recently, Boc et al.^22^ did in vitro testing of a dry powder aerosol formulation of Survanta®, a clinical bovine-derived lung surfactant, whereas Bianco et al.^23^ investigated aerosol delivery with a vibrating mesh nebulizer of the porcine surfactant suspension Curosurf® in spontaneously breathing surfactant-deficient rabbits supported with nasal CPAP. The latter study demonstrated a similar improvement in oxygenation and ventilation efficacy index compared to intratracheal surfactant administration. These findings are in line with our studies using fully synthetic surfactant formulations^14,24^. The increased interest in aerosol delivery of surfactant and medications to premature infants supported with noninvasive ventilation reflects an unmet need in clinical neonatology and may further improve outcome of this fragile population.

In summary, surface activity, chemical stability, and aerosol and physical properties are in favor of dry powder lung surfactant with 49% DPPC, 21% POPG, 3% B-YL, 25% Trehalose, and 2% NaCl as a leading candidate for potential clinical use in noninvasive synthetic lung surfactant administration. Preclinical studies in surfactant-deficient animal models will have to confirm whether these in vitro findings hold true in vivo.

## Data Availability

All data generated or analyzed during this study are included in this article.
